# Ginsenoside Rg1 relieves rat intervertebral disc degeneration and inhibits IL-1β-induced nucleus pulposus cell apoptosis and inflammation via NF-κB signaling pathway

**DOI:** 10.1007/s11626-024-00883-6

**Published:** 2024-03-14

**Authors:** Lei Yu, Ying-Jie Hao, Zhi-Nan Ren, Guang-Duo Zhu, Wei-Wei Zhou, Xu Lian, Xue-Jian Wu

**Affiliations:** Department of Orthopedics, The First Affiliated Hospital of Zhengzhou University, No. 50 Jianshe East Road, Erqi District, Zhengzhou, 450001 Henan Province China

**Keywords:** Ginsenoside Rg1, Intervertebral disc degeneration, Nucleus pulposus cells, Inflammatory response, Extracellular matrix, NF-κB pathway

## Abstract

The study aimed to investigate the effect of ginsenoside Rg1 on intervertebral disc degeneration (IVDD) in rats and IL-1β-induced nucleus pulposus (NP) cells, and explore its underlying mechanism. Forty IVDD rat models were divided into the IVDD group, low-dose (L-Rg1) group (intraperitoneal injection of 20 mg/kg/d ginsenoside Rg1), medium-dose (M-Rg1) group (intraperitoneal injection of 40 mg/kg/d ginsenoside Rg1), and high-dose (H-Rg1) group (intraperitoneal injection of 80 mg/kg/d ginsenoside Rg1). The pathological change was observed by HE and safranin O-fast green staining. The expression of IL-1β, IL-6, TNF-α, MMP3, aggrecan, and collagen II was detected. The expression of NF-κB p65 in IVD tissues was detected. Rat NP cells were induced by IL-1β to simulate IVDD environment and divided into the control group, IL-1β group, and 20, 50, and 100 µmol/L Rg1 groups. The cell proliferation activity, the apoptosis, and the expression of IL-6, TNF-α, MMP3, aggrecan, collagen II, and NF-κB pathway–related protein were detected. In IVDD rats, ginsenoside Rg1 improved the pathology of IVD tissues; suppressed the expression of IL-1β, IL-6, TNF-α, aggrecan, and collagen II; and inhibited the expression of p-p65/p65 and nuclear translocation of p65, to alleviate the IVDD progression. In the IL-1β-induced NP cells, ginsenoside Rg1 also improved the cell proliferation and inhibited the apoptosis and the expression of IL-6, TNF-α, aggrecan, collagen II, p-p65/p65, and IκK in a dose-dependent manner. Ginsenoside Rg1 alleviated IVDD in rats and inhibited apoptosis, inflammatory response, and ECM degradation in IL-1β-induced NP cells. And Rg1 may exert its effect via inhibiting the activation of NF-κB signaling pathway.

**Supplementary Information:**

The online version contains supplementary material available at 10.1007/s11626-024-00883-6.

## Introduction

Intervertebral disc (IVD) is an important component of the spinal structure, which is composed of a gel-like nucleus pulposus (NP) surrounded by a fibrocartilaginous annulus fibrosus, and cartilage endplates (Ashinsky *et al*. 2021). IVD degeneration (IVDD) is the main cause of disc herniation, discogenic pain, and other disc degeneration–related diseases (Risbud and Shapiro 2014). As a common, complex, and multifactorial orthopedic disease, IVDD is mainly manifested as the apoptosis and necrosis of NP cells, and imbalance of extracellular matrix (ECM) degradation and synthesis, seriously threatening the patients’ life and social economy (Kos *et al*. 2019). There are many factors of IVDD, such as genetics, mechanical stress, oxidative stress, and inflammatory response (Xin *et al*. 2022). However, the pathogenesis of IVDD has not been elucidated. At present, the clinical methods, such as surgery and drug therapy, for IVDD only temporarily alleviate the pain and cannot solve the problem fundamentally. So, it is particularly important to find new drugs or treatments for inhibiting NP cell apoptosis and ECM degradation, to potentially cure IVDD.

Ginsenoside Rg1 is the main active component of ginseng, which has a wide range of pharmacological effects on the central nervous system, cardiovascular system, and endocrine system, and has been proven to have neuroprotective effect and anti-inflammatory and anti-apoptotic properties (Alolga *et al*. 2020, Gao *et al*. 2020, Sun *et al.* 2022). For example, ginsenoside Rg1 had been reported to promote cerebral angiogenesis via up-regulating VEGF expression through PI3K/Akt/mTOR signaling pathway after ischemic stroke (Chen *et al*. 2019). Luo *et al*. (2020) found that ginsenoside Rg1 attenuated LPS-induced inflammation and apoptosis in neonatal rat cardiomyocytes and restored impaired cardiac function in septic mice via blocking the TLR4/NF-κB/NLRP3 pathway. He *et al*. (2021) suggested that ginsenoside Rg1 improved pathological damages in the ovary and uterus by increasing anti-oxidant and anti-inflammatory abilities while reducing the expression of senescence signaling pathways in premature ovarian insufficiency mouse models. Furthermore, the previous study demonstrated that ginsenoside Rg1 promoted NP cell proliferation to improve IVDD by regulating Wnt/β-catenin pathway (Yu *et al*. 2020). And Yang *et al*. (2022) indicated that ginsenoside Rg1 had a significant inhibitory effect on the secretion level of inflammatory factors, redox activity, ECM degradation, and cell apoptosis both in IVD tissue and NP cells, by suppressing the activation of YAP1/TAZ signaling pathway. However, studies could not comprehensively reveal the mechanism of ginsenoside Rg1 on IVDD.

Therefore, this study aimed to explore the effect of ginsenoside Rg1 on the apoptosis, ECM degradation, and inflammatory response in IL-1β-induced NP cells in vitro, and to investigate the possible mechanism: whether ginsenoside Rg1 regulated the NF-κB signaling pathway, to mediate the apoptosis, ECM degradation, and inflammatory response in IL-1β-induced NP cells, which might lay a certain theoretical foundation and provide a new direction for the treatment of IVDD.

## Materials and methods

### Animal models

All experiments were approved by the Ethics Committee of Zhengzhou University (2023-KY-0305-002). Fifty male Sprague-Dawley rats (8 wk) weighting 200–250 g were purchased from Shanghai Slac Laboratory Animal Co., Ltd. (Shanghai, China) (SCXKI (Hu) 2017-0005). All rats were maintained 1 wk to understand specific pathogen-free conditions on a 12-h light/cycle with temperature 20–25℃ and free access to food and water. Forty-two rats were randomly selected to construct models of IVDD according to Zhao *et al*. (2021). Briefly, the rats were anesthetized 3% sodium pentobarbital (30 mg/kg). The IVDD rat models were constructed by surgically excising the paraspinal musculature and the supraspinous and interspinous ligaments from neck 2–7 (C2–C7). Specific operation steps can be found in the previous research (Yu *et al*. 2020). There were two rats sacrificed during modeling. The other eight rats were treated with sham surgery as the control group.

Ginsenoside Rg1 (> 98%) were purchased from the National Institutes for Food and Drug Control  (Beijing, China). A total of 40 rats with cervical IVDD were successfully constructed, and randomly divided into four groups (10 rats): IVDD group (same amount of saline), low-dose (L-Rg1) group (intraperitoneal injection of 20 mg/kg/d ginsenoside Rg1), medium-dose (M-Rg1) group (intraperitoneal injection of 40 mg/kg/d ginsenoside Rg1), and high-dose (H-Rg1) group (intraperitoneal injection of 80 mg/kg/d ginsenoside Rg1). After 8 wk of administration, all of the rats were euthanized, and the IVD tissues were collected for the subsequent experiments.

### Hematoxylin-eosin (HE) staining

Firstly, the soft tissues surrounding the IVD tissue were removed. Then, the IVD tissue of rats was fixed in 4% paraformaldehyde for 48 h, and decalcified in 15% ethylenediaminetetraacetic acid (EDTA) solution. After dehydrated with gradient ethanol, the tissues were embedded whole in paraffin and cut into the slices of thickness 5 μm. After deparaffinization, the sections were stained with hematoxylin for 5 min staining and eosin solution for 30 s. Then, the sections were dehydrated with ethanol, cleared with xylene, and sealed with neutral resin. The morphology of IVD tissues was observed under a microscope (Olympus, Tokyo, Japan), with at least three sections for each rat.

### Safranin O-fast green staining

The IVD samples embedded in paraffin were cut into sections of 5 μm as described previously: After deparaffinization, the sections were stained with the Weigert solution for 3 min. After rinsing with tap water for 10 min, the sections were stained with fast green for 3 min, and safranin O for 5 min. Sections were mounted with neural resin after they were rinsed with ethanol and xylene. The morphology of IVD tissues was observed under a microscope (Olympus), with at least three sections for each rat. And the degree of cartilage degeneration was determined by the modified Mankin score, according to Go *et al*. (2022). Three visual fields were selected to calculate the averaged Mankin score for each section.

### Immunofluorescence

After being dewaxed and rehydrated, the sections were placed in boiling citric acid buffer for 10 min to retrieve the antigen and then incubated in 3% H_2_O_2_ at 37℃ for 15 min to block endogenous peroxidase activity. After being blocked with 5% goat serum for 1 h, the sections were incubated with primary antibody against NF-κB p65 (1:200, Abcam, Waltham, MA) at 4℃ overnight. And the secondary antibody (FITC-conjugated) was incubated at 37℃ for 1 h. The sections were mounted with antifade mounting medium with DAPI. And the sections, at least three sections for each rat, were observed and the fluorescent images were obtained using a fluorescence microscope (DM2500, Leica, Wetzlar, Germany).

### Rat NP cell isolation, culture, and identification

According to the previous study (Yu *et al*. 2020), SD rats were euthanized with 3% pentobarbital sodium. After removing the cervical skin, the cervical intervertebral disc of the rats was separated under the anatomical microscope, and immersed and disinfected in 75% ethanol for 5–10 min. Then, the gel-like NP tissue from the cervical intervertebral disc was separated and washed with sterile PBS twice. Then, NP tissues were cut into small fragments, and released and isolated with 0.25% trypsin for 10 min and 0.2% collagenase II for 5 h at 37℃ in DMEM/F12 containing 0.1% of fetal bovine serum and 1% of penicillin-streptomycin. After filtration by sterile cell sieves, the cells were washed with PBS and collected by centrifugation (1000 r/min for 5 min).

After isolation, NP cells were resuspended in the DMEM/F12 medium with 10% FBS (Gibco, Billings, MT), and 1% penicillin-streptomycin and cultured in an incubator at 37℃ with 5% CO_2_. The medium was changed every 3 d. The NP cells from 2 to 3 generations were used for the following experiments. And the morphology of rat NP cells was observed under an inverted microscope and identified by toluidine blue staining.

### Experimental groups

After 2–3 passages, NP cells were seeded in 6-well plates and divided into five groups. Different experimental groups were given different treatments. The control group is the group without any treatment; for the IL-1β group, NP cells were stimulated with IL-1β (10 ng/mL) for 24 h to simulate IVDD environment (Huang *et al*. 2022). For the 20 µmol/L Rg1 group, NP cells were co-treated with IL-1β and 20 µmol/L ginsenoside Rg1 in the DMEM/F12 medium. For the 50 µmol/L Rg1 group, NP cells were co-treated with IL-1β and 50 µmol/L ginsenoside Rg1 in the DMEM/F12 medium. For the 100 µmol/L ginsenoside Rg1 group, NP cells were co-treated with IL-1β and 100 µmol/L Rg1 in the DMEM/F12 medium. After 24 h of culture at 37℃ with 5% CO_2_, further experiments in cells were conducted.

### Cell counting kit-8 (CCK-8) assay

CCK-8 assay (Solarbio, Beijing, China) was used to determine the effect of Rg1 on the cell viability of NP cells. For the detection of ginsenoside Rg1 and IL-1β cytotoxicity, the NP cells with or without IL-1β induced were seeded in a 96-well plate (5 × 10^3^/well) with three replicate wells per group and intervened by various concentrations of ginsenoside Rg1 (0, 10, 20, 50, 100, 200 μmol/L). After being cultured for 24 h, the cells in each well were incubated with CCK-8 solutions for 4 h at 37℃. Then, the absorbance was measured at 450 nm on an automatic microplate reader (ELX80, Bio-Tek, Winooski, VT). As for the cell proliferation, the cells were seeded in a 96-well plate (2 × 10^4^/well) with three replicate wells per group and cultured for 24 h, 48 h, and 72 h. Then, 10 μL of the CCK-8 solution was added in each well, and incubated at 37℃ for 4 h. The absorbance was checked as above. The experiment was repeated three times. And the proliferation curve was plotted with time point as the *X*-axis and *A* value as the *Y*-axis.

### Flow cytometry assay

To analyze the effect of ginsenoside Rg1 on apoptosis, Annexin V/PI double staining by flow cytometric analyses was performed. Briefly, NP cells were incubated in 6-well plates at 5 × 10^4^/well and cultured for 48 h after treatment with drugs. Then, NP cells were collected by routine digestion and centrifugation, and resuspended by binding buffer solution, and then subsequently stained with Annexin V-FITC and PI according to the protocols of Annexin V/PI Apoptosis Detection Kits (Solarbio, Beijing, China). Then, the cell apoptosis was assessed by FACScan flow cytometry (BD Biosciences, Franklin Lakes, NJ). Total apoptotic rate = early apoptotic rate + late apoptotic rate. The experiment was repeated three times.

### Quantitative real-time PCR (qPCR)

The total RNA from NP tissues and NP cells of each group was isolated using TRIzol (Invitrogen). Then, cDNA was synthetized by a reverse transcriptase kit (TaKaRa, Kyoto, Japan) according to the manufacturer’s protocols. qRT-PCR detection was conducted on 7900 HT Fasting Real-Time PCR System (Applied Biosystems) with SYBR Green Mix Kit and primers of IL-1β, IL-6, TNF-α, aggrecan, collagen II, MMP3, and GAPDH. The relative expression was quantitated using the 2^−ΔΔCt^ method, with GAPDH as normalized reference. The experiment was repeated three times.

### ELISA

The contents of IL-1β, TNF-α, and IL-6 in NP tissues and NP cells were detected by ELISA kits (Solarbio, Beijing, China). The operation was performed in strict accordance with the ELISA kit instructions. The experiment was repeated three times.

### Western blot

The NP tissues and NP cells cultured of 24 h were collected in each group. The total protein was extracted by cell lysis, and the protein concentration was determined by the BCA method. For immunoblotting, protein samples were separated by SDS-PAGE, and transferred into PVDF membranes (Merck Millipore, Billerica, MA). The membranes were blocked with 5% skim milk in TBST solution at room temperature for 2 h. The primary antibodies were incubated with different membranes at 4℃ overnight. The secondary antibodies, HRP-conjugated goat anti-rabbit or goat anti-mouse IgG, were incubated for 1 h. The immunoreactive proteins were visualized by electro-chemiluminescence detection system and the relation expression was calculated using ImageJ with GAPDH as the internal reference. The experiment was repeated three times.

### Statistical analysis

All experiments were executed independently and repeated at least three times. All results were analyzed using statistic software SPSS 21.0. The data were demonstrated as means ± standard deviation, and Student’s *t* test or one-way analysis of variance (ANOVA) analysis was used to determine the significant differences between two groups or among multiple groups. *p* < 0.05 was considered statistically significant.

## Results

### Ginsenoside Rg1 improved the pathology of IVD tissues in IVDD rats

**Figure 1.**
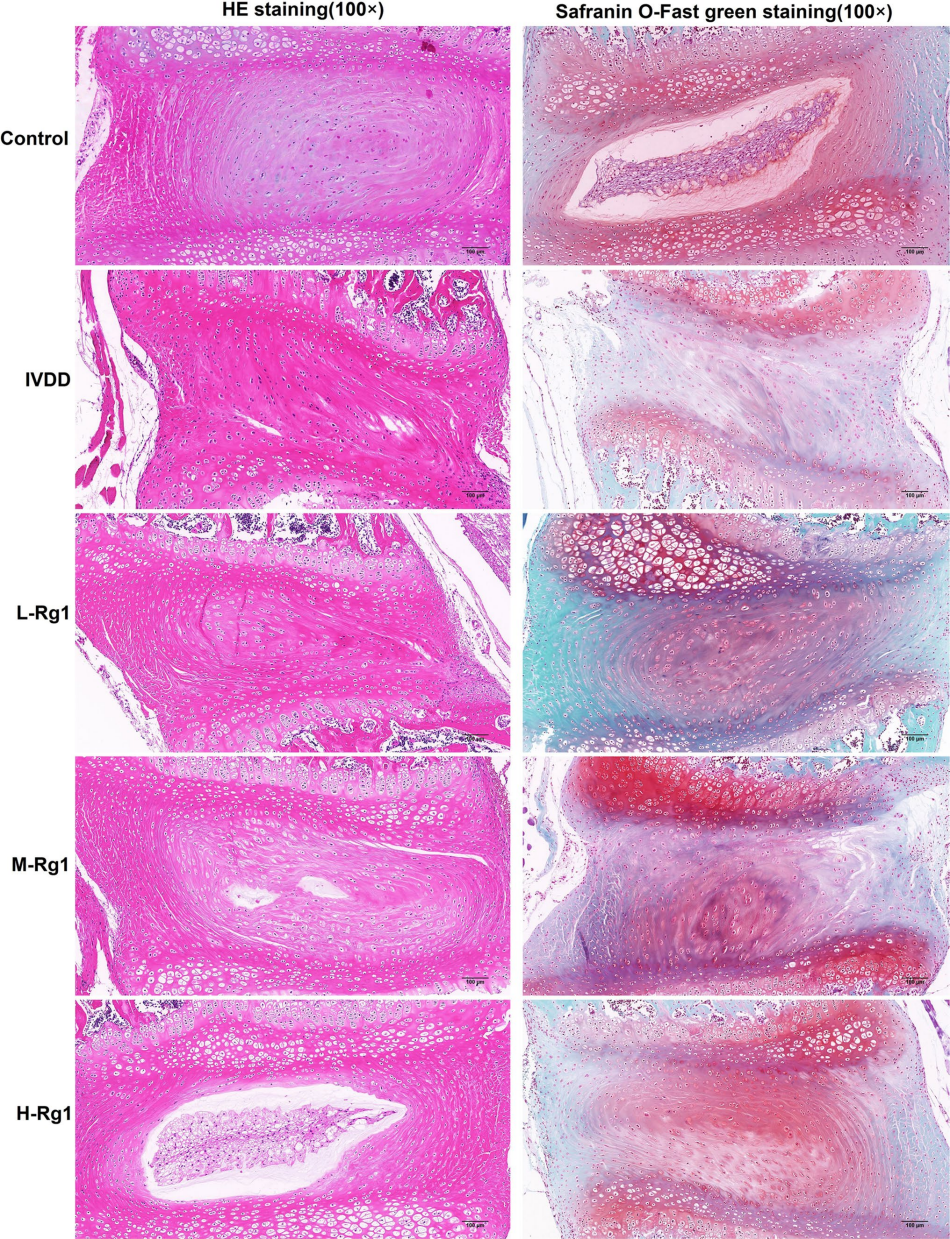
Effect of ginsenoside Rg1 on pathology of IVD tissues in IVDD rats. Control group: *n* = 8; IVDD group: *n* = 10; L-Rg1 group: *n* = 10; M-Rg1 group: *n* = 10; H-Rg1 group: *n* = 10. *Magnification*, 100×; *scale bar*, 100 μm.

To observe the effect of ginsenoside Rg1 on the progression of IVDD, the histological staining of IVD tissues in IVDD rats was performed by HE staining and safranin O-fast green staining (Fig. 1). The IVD tissues in IVDD rats presented with varying degrees of loss in cartilage matrix and varying levels of damage of IVD tissue. Compared with the normal group, the numbers of NP cells were decreased and the boundary between the NP and AF was interrupted and the collagen fiber was arranged disorderly in IVDD rats. However, the IVD tissue was improved to varying degrees after the administration of ginsenoside Rg1, and the improvement was positively correlated with the concentration of ginsenoside Rg1. In all groups, the modified Mankin score in the IVDD group was 9.60 ± 2.33, significantly higher than that in the control group (0.25 ± 0.43), while the modified Mankin scores in the L-Rg1, M-Rg1, and H-Rg1 groups were 7.70 ± 1.42, 5.70 ± 1.10, and 2.80 ± 1.60, respectively, lower than those in the IVDD group (Supplemental Figures). The data indicated that ginsenoside Rg1 alleviated the progression of IVDD.

### Ginsenoside Rg1 inhibited the inflammatory response in IVDD rats

**Figure 2.**
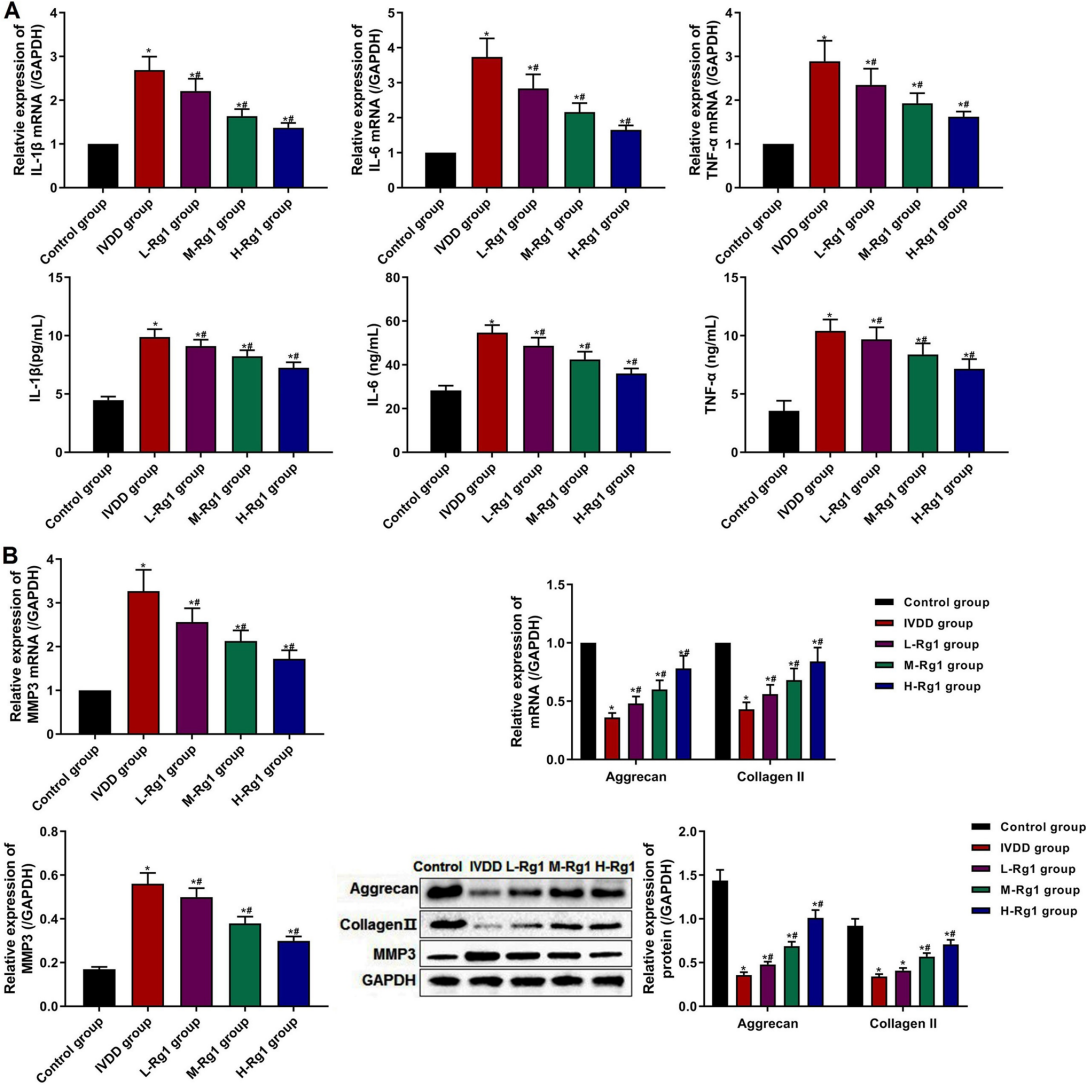
Effect of ginsenoside Rg1 on inflammatory factors and ECM-related proteins in IVDD rats. (***A***) The levels of IL-1β, IL-6, and TNF-α in IVD tissues were examined by qRT-PCR and ELISA. (***B***) The expression of MMP3, aggrecan, and collagen II was examined by qRT-PCR and Western blot. Data were presented as mean ± SD from each rat (control group: *n* = 8; IVDD group: *n* = 10; L-Rg1 group: *n* = 10; M-Rg1 group: *n* = 10; H-Rg1 group: *n* = 10). *p < 0.05, vs control group; ^#^*p* < 0.05, vs IVDD group.

IVDD is characterized by increased levels of the proinflammatory cytokines, IL-1β, IL-6, and TNF-α (Francisco *et al*. 2023). So, the secretion levels of inflammatory factors were examined by qRT-PCR and ELISA to further determine whether ginsenoside Rg1 could reduce the inflammatory response in IVDD rats (Fig. 2*A*). Compared with the control group, the levels of IL-1β, IL-6, and TNF-α increased in the IVDD group, while the levels of IL-1β, IL-6, and TNF-α in the L-Rg1, M-Rg1, and H-Rg1 groups were lower than those in the IVDD group, and inflammatory factors were decreased with the concentration of ginsenoside Rg1. The data suggested that ginsenoside Rg1 significantly reduced the inflammatory response in IVDD rats.

### Ginsenoside Rg1 suppressed the ECM degradation in NP tissues in IVDD rats

Matrix metalloproteinases (MMP) is a major matrix-degrading enzyme involved in IVDD, and it could promote the degradation of ECM molecules, such as aggrecan and collagen II (Chen *et al*. 2023). Therefore, the effect of ginsenoside Rg1 on ECM degradation was examined. qRT-PCR and Western blot results (Fig. 2*B*) showed that the expression of MMP3 was increased, while the expression of aggrecan and collagen II was decreased in the NP tissues in the IVDD group, compared with the control group. By contrast, compared with the IVDD group, the expression of MMP3 was notably reduced and the expression of aggrecan and collagen II was increased in the Rg1 groups, in a dose-dependent manner, suggesting that ginsenoside Rg1 significantly suppressed the ECM degradation in NP tissues in IVDD rats.

### Ginsenoside Rg1 inhibited the activation of NF-κB signaling pathway in IVDD rats

**Figure 3.**
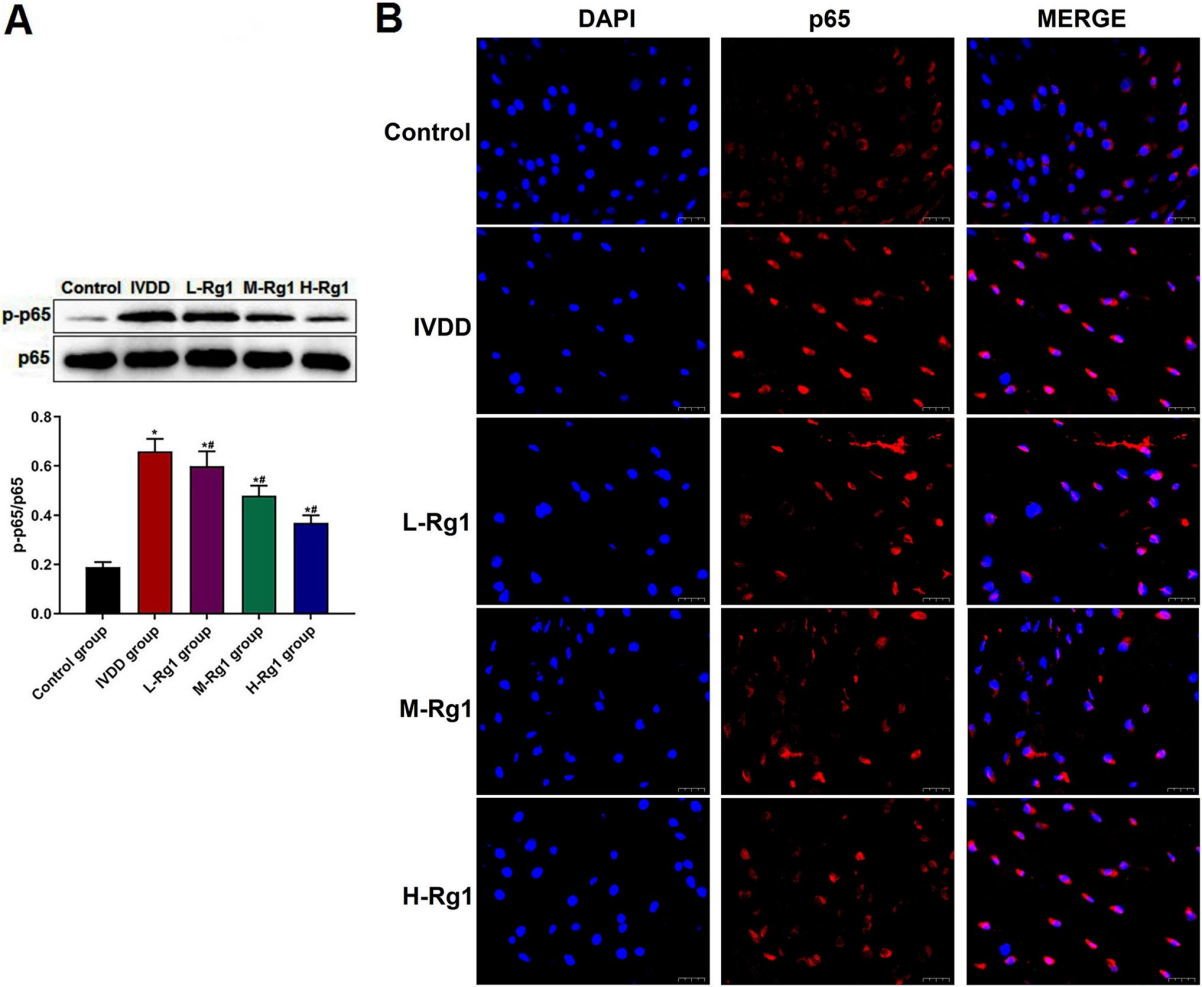
Effect of ginsenoside Rg1 on NF-κB signaling pathway in IVDD rats. (***A***) The expression of NF-κB p-p65 was detected by Western blot; *p < 0.05, vs control group; ^#^*p* < 0.05, vs IVDD group. (***B***) The nuclear translocation of p65 was examined by immunofluorescence assay. Data were presented as mean ± SD from each rat (control group: *n* = 8; IVDD group: *n* = 10; L-Rg1 group: *n* = 10; M-Rg1 group: *n* = 10; H-Rg1 group: *n* = 10). Magnification, 400×; *scale bar*, 20 μm.

NF-κB signaling pathway plays a crucial role in the inflammatory and IVDD progression (Zhang *et al*. 2021a). So, the effect of ginsenoside Rg1 on the NF-κB signaling pathway in IVDD rats was examined by Western blot and immunofluorescence assay. The ratio of p-p65/p65 in the NP tissues was up-regulated in the IVDD group, compared with the control group, while the ratio of p-p65/p65 was down-regulated in the Rg1 groups, in a dose-dependent manner (Fig. 3*A*). Immunofluorescence assay (Fig. 3*B*) showed that compared to the control group, p65 was translocated into the nucleus in NP tissues in the IVDD group. However, the nuclear translocation of p65 in the L-Rg1, M-Rg1, and H-Rg1 groups was significantly suppressed, compared with the IVDD group. The above results suggested that ginsenoside Rg1 inhibited the activation of NF-κB signaling pathway in the NP tissues in IVDD rats.

### Ginsenoside Rg1 promoted the proliferation of IL-1β-induced NP cells

**Figure 4.**
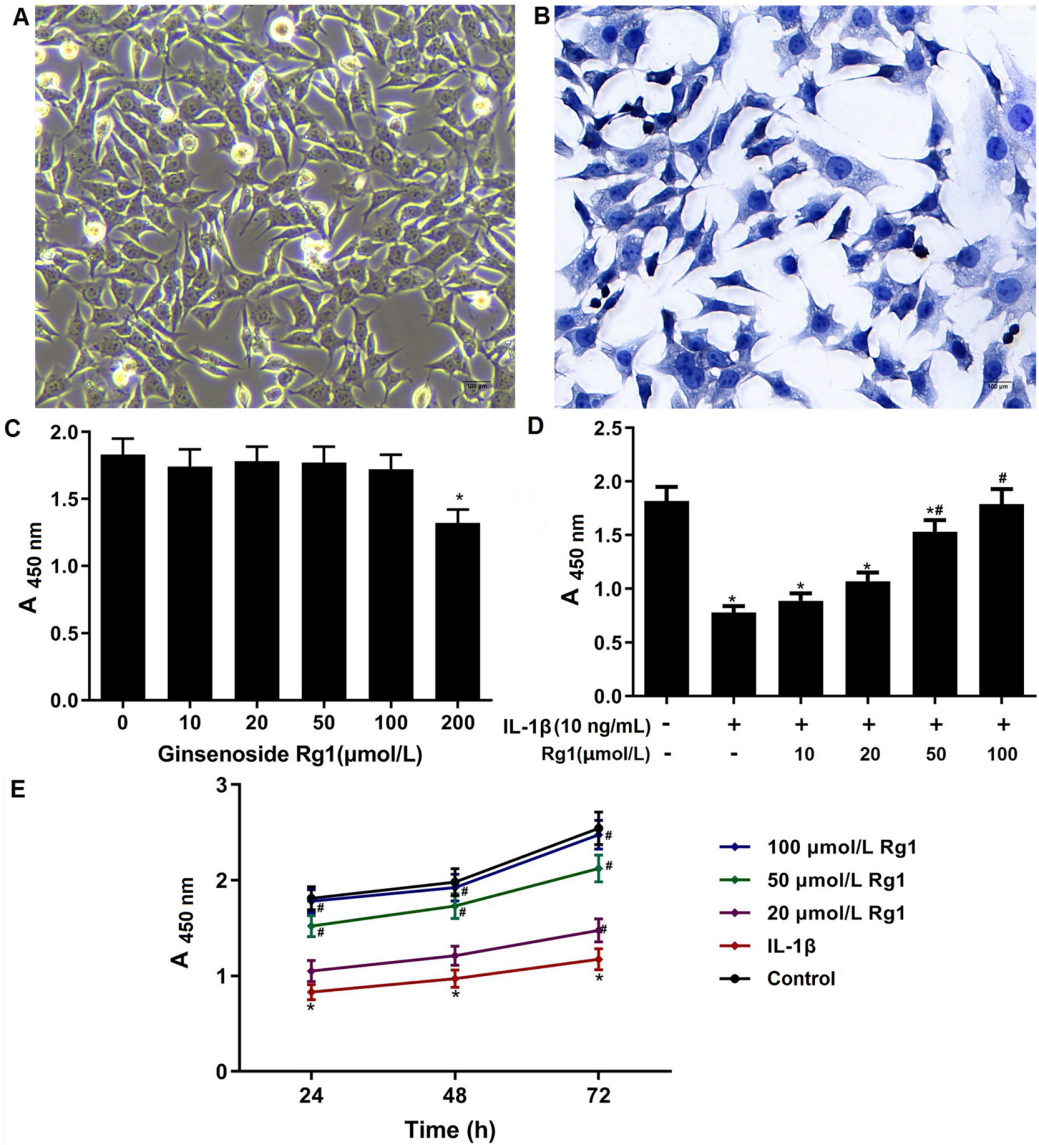
Effect of ginsenoside Rg1 on proliferation of IL-1β-induced NP cells. (***A***) The morphology of NP cells observed under a light microscope; magnification, 200×; *scale bar*, 100 μm. (***B***) The morphology of NP cells identified by toluidine blue staining. (***C***) The viability of NP cells treated with various concentrations of ginsenoside Rg1 (0, 10, 20, 50, 100, 200 μmol/L); *p < 0.05, vs 0 μmol/L ginsenoside Rg1. (***D***) The viability of NP cells treated with IL-1β (10 ng/mL) and various concentrations (0, 10, 20, 50, 100 μmol/L) of ginsenoside Rg1; *p < 0.05, vs normal NP cells; ^#^*p* < 0.05, vs NP cells induced by IL-1β (10 ng/mL). (***E***) The proliferation of NP cells in each group detected by CCK-8 assay. Data were presented as mean ± SD of at least three repeated experiments. *p < 0.05, vs control group; ^#^*p* < 0.05, vs IL-1β group.

Firstly, the morphology of NP cells was observed under a light microscope and the cells were identified by toluidine blue staining, as shown in Fig. 4*A* and *B*.

To evaluate the cytotoxic effect of ginsenoside Rg1 on NP cells, the NP cells were treated with various concentrations (0, 10, 20, 50, 100, 200 μmol/L) of ginsenoside Rg1 for 24 h. The CCK-8 assay showed that ginsenoside Rg1 at a concentration of 100 μmol/L or less had no obvious cytotoxic effect on NP cells, compared to the untreated cells, while 200 μmol/L ginsenoside Rg1 significantly inhibited the viability of the NP cells (Fig. 4*C*). So, ginsenoside Rg1 at concentrations of 20, 50, and 100 μmol/L was applied to the subsequent experiments.

To simulate the microenvironment of NP cells in the IVDD, IL-1β (10 ng/mL) was used to stimulate NP cells *in vitro*. As shown in Fig. 4*D*, Il-1β significantly decreased the viability of NP cells, while ginsenoside Rg1 could dose-dependently alleviate the toxicity of IL-1β to NP cells. When the concentration of ginsenoside Rg1 was 100 μmol/L, it could completely alleviate the toxicity of IL-1β to NP cells.

CCK-8 assay was performed to detect the effect of ginsenoside Rg1 on the proliferation activity of IL-1β-induced NP cells (Fig. 4*E*). Compared with the control group, the cell proliferation activity of NP cells in the IL-1β group was obviously decreased. At 72 h, the cell proliferation activity in the 20 µmol/L Rg1 group was significantly higher than that in the IL-1β group. As well as the cell proliferation activities in the 50 µmol/L Rg1 group and 100 µmol/L Rg1 group were higher than those in the IL-1β group, at 24 h, 48 h, and 72 h. The results indicated that ginsenoside Rg1 promoted the proliferation of IL-1β-induced NP cells in a dose-dependent manner.

### Ginsenoside Rg1 inhibited the apoptosis of IL-1β-induced NP cells

**Figure 5.**
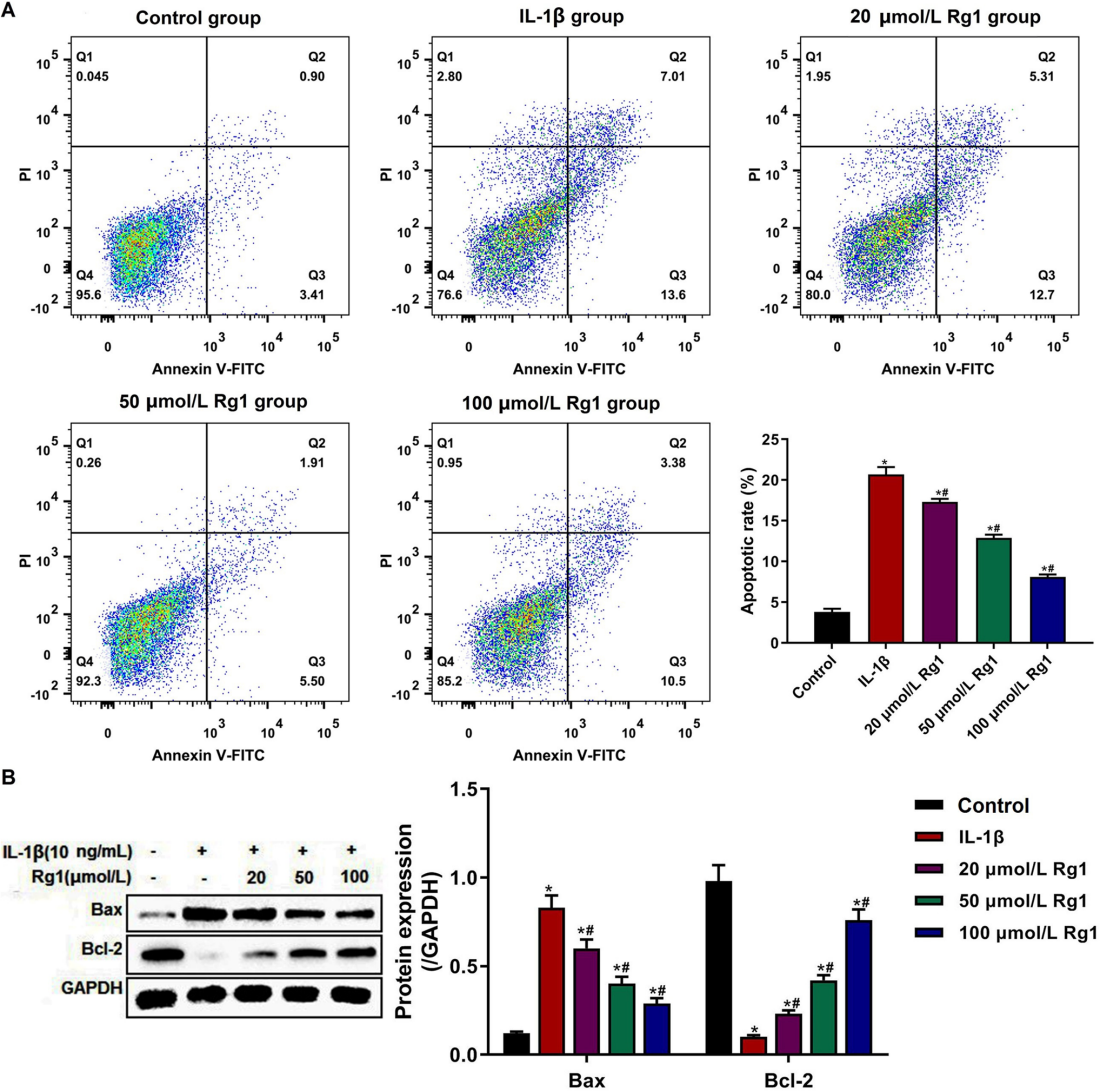
Effect of ginsenoside Rg1 on apoptosis of IL-1β-induced NP cells. (***A***) The apoptosis of IL-1β-induced NP cells detected by flow cytometry. (***B***) The expression of apoptosis-related protein Bax and Bcl-1 detected by Western blot. Data were presented as mean ± SD of at least three repeated experiments. *p < 0.05, vs control group; ^#^*p* < 0.05, vs IL-1β group.

Abnormal apoptosis of NP cells also contributes to the progression of IVDD (Zhu *et al*. 2022). So, the apoptosis of NP cells treated with or without IL-1β or/and ginsenoside Rg1 was detected. FCM analysis indicated that the apoptotic rate in the IL-1β group was higher than that in the control group. The apoptotic rate in the 20, 50, and 100 µmol/L Rg1 groups was lower than that in the IL-1β group, and changes in the 100 µmol/L Rg1 group were most marked (Fig. 5*A*). Furthermore, the downstream marker proteins were detected by Western blot analysis (Fig. 5*B*). Compared with the control group, the expression of Bax was significantly up-regulated and the expression of Bcl-2 was significantly down-regulated in the IL-1β group, while ginsenoside Rg1 could dose-dependently inhibit the effect of IL-1β on the expression of Bax and Bcl-2 in NP cells. The results indicated that ginsenoside Rg1 inhibited the apoptosis of IL-1β-induced NP cells in a dose-dependent manner.

### Ginsenoside Rg1 suppressed inflammatory response in IL-1β-induced NP cells

**Figure 6.**
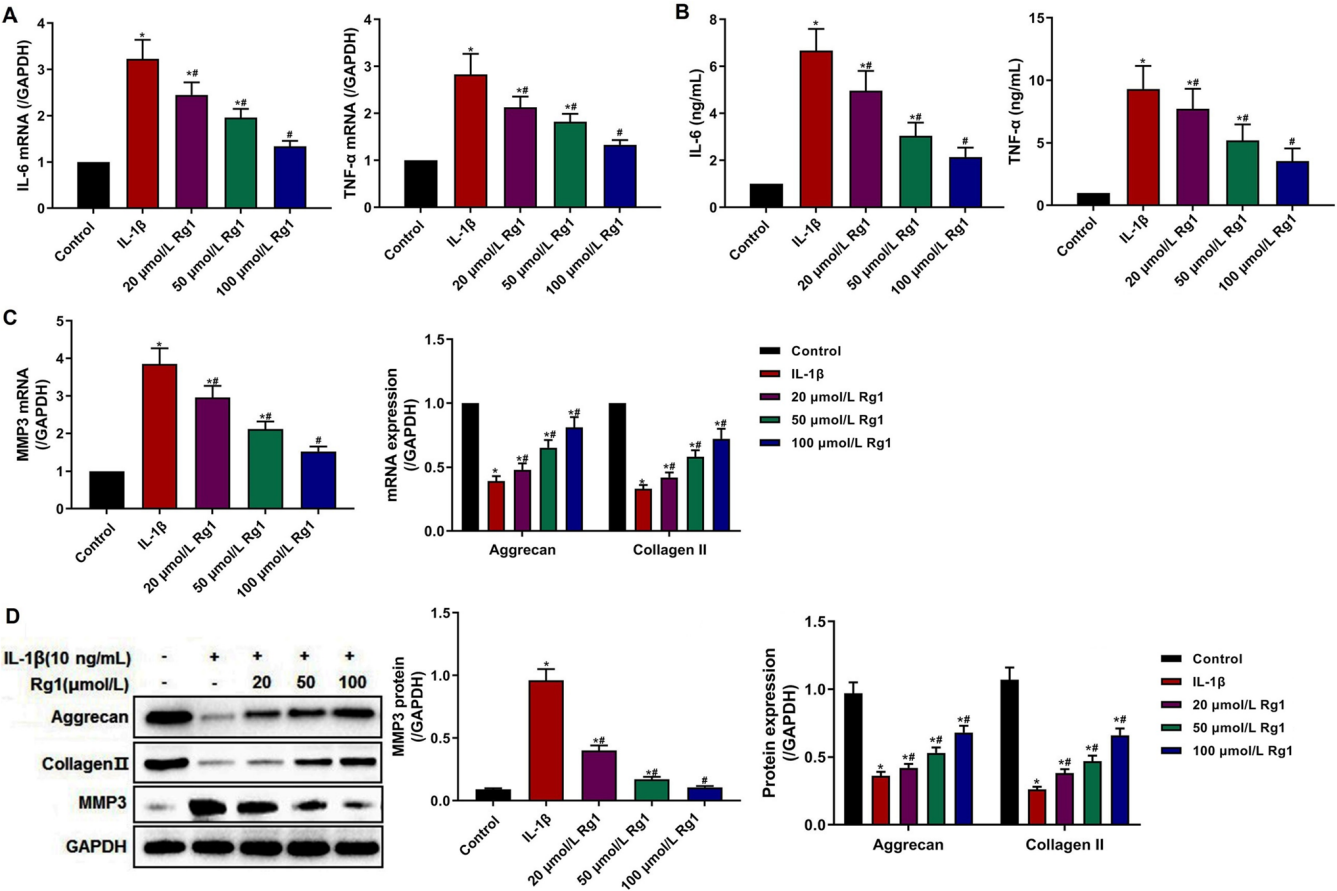
Effect of ginsenoside Rg1 on inflammatory factors and ECM-related protein in IL-1β-induced NP cells. (***A*, *B***) The expression of IL-6 and TNF-α detected by qRT-PCR and ELISA. (***C***, ***D***) The relative expression of MMP3, aggrecan, and collagen II detected by qRT-PCR and Western blot. Data were presented as mean ± SD of at least three repeated experiments. *p < 0.05, vs control group; ^#^*p* < 0.05, vs IL-1β group.

There is accumulating evidence that inflammation is closely related to IVDD (Khan *et al*. 2017). Therefore, the mRNA and protein of the inflammatory factors, TNF-α and IL-6, were evaluated by qRT-PCR and ELISA assay (Fig. 6*A*, *B*). The expression of TNF-α and IL-6 in the IL-1β group was higher than that in the control group, while the expression and concentration of TNF-α and IL-6 were increased with the increase of ginsenoside Rg1 concentration in the 20, 50, and 100 µmol/L Rg1groups, higher than those in the IL-1β group. The results suggested that ginsenoside Rg1 suppressed the expression of inflammatory factors in IL-1β-induced NP cells.

### Ginsenoside Rg1 attenuated ECM degradation in IL-1β-induced NP cells

Catabolic enzymes, such as MMP3, are closely related to the ECM catabolism of NP cells (Wang *et al*. 2023). So, the expression of MMP3 and ECM-related proteins (aggrecan and collagen II) was detected by qRT-PCR and Western blot analysis to assess the catabolic activity of the NP cells. As shown in Fig. 6*C* and *D*, the expression of MMP3 was up-regulated, and the expression of aggrecan and collagen II was reduced in the IL-1β group. And compared with the IL-1β group, the expression of MMP3 was decreased, and the expression of aggrecan and collagen II was significantly increased in the 20, 50, and 100 µmol/L Rg1 groups, and the changes in the 100 µmol/L Rg1 group were more obvious. The above results suggested that ginsenoside Rg1 inhibited the catabolic activity of ECM, and promoted the expression of aggrecan and collagen II, the major components of ECM, to attenuate the degradation of ECM in IL-1β-induced NP cells.

### Ginsenoside Rg1 inhibited the activation of NF-κB signaling pathway in IL-1β-induced NP cells

**Figure 7.**
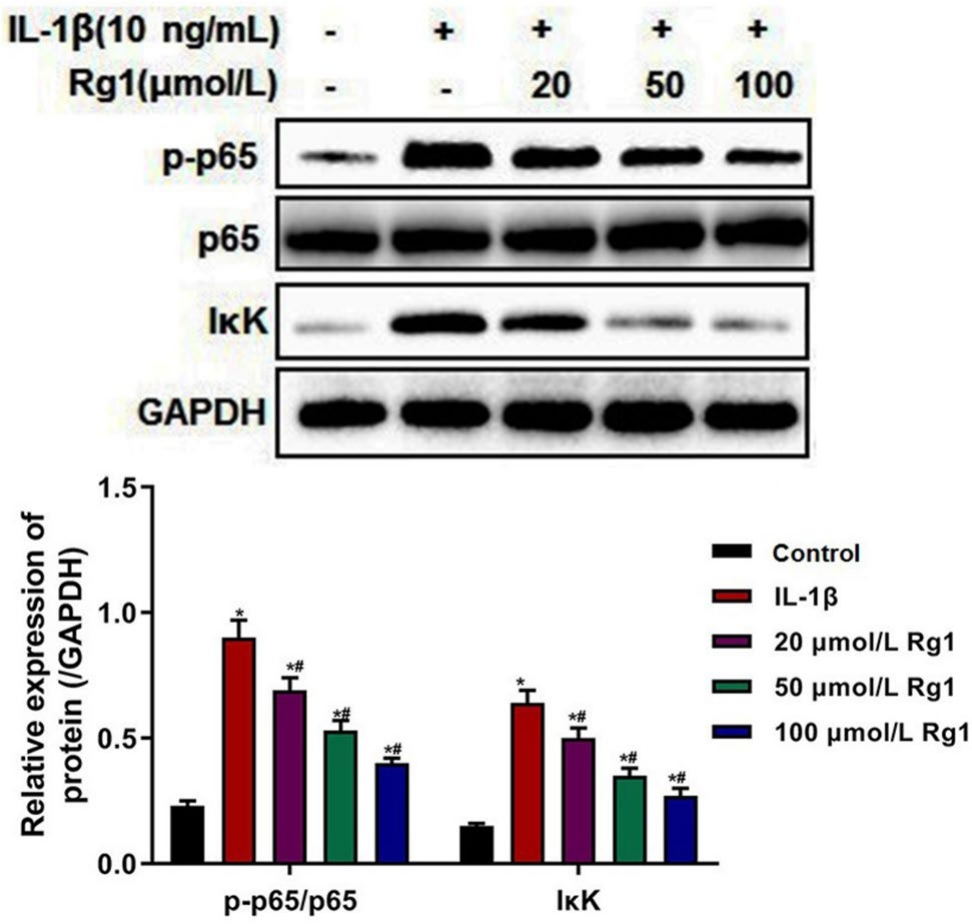
Effect of ginsenoside Rg1 on the NF-κB signaling pathway in IL-1β-induced NP cells. The relative expression of NF-κB p-p65 and IκK detected by Western blot. Data were presented as mean ± SD of at least three repeated experiments. *p < 0.05, vs control group; ^#^*p* < 0.05, vs IL-1β group.

It is well known that the NF-κB signaling pathway plays an important role in a variety of biological processes, such as cellular immunity, inflammation, growth, and apoptosis (Jimi *et al*. 2019). And NF-κB signaling pathway has been regarded as a pathogenic factor in the process of IVDD (Zhang *et al*. 2021a). So, the effects of ginsenoside Rg1 on NF-κB signaling pathway in NP cells were measured. As shown in Fig. 7, the expression of IκK and p-p65/p65 in the IL-1β group was significantly higher than that in the control group. And compared with the IL-1β group, the expression of IκK and p-p65/p65 in the 20, 50, and 100 µmol/L Rg1 groups was significantly decreased, especially in the 100 µmol/L Rg1 group. The results indicated that NF-κB signaling pathway was activated in the IL-1β-induced NP cells, and ginsenoside Rg1 inhibited the activation of NF-κB signaling pathway in the IL-1β-induced NP cells.

The above results indicated that ginsenoside Rg1 mediated cell apoptosis, ECM degradation, and inflammation in IL-1β-induced NP cells probably by regulating the NF-κB signaling pathway.

## Discussion

Studies have suggested that inflammation is an essential characteristic in NP tissues during the pathological process of IVDD. And inflammatory cytokines (IL-1β and TNF-α) were closely related to various pathological IVDD processes, including inflammatory response, matrix destruction, and apoptosis (Wang *et al*. 2020). In the healthy NP tissue, the NP cells play an important role in the metabolic balance of ECM, including aggrecan and collagen (Jiang *et al*. 2021). Under the pathological status, NP tissues can be influenced by external factors, such as mechanical stress, inflammation, and the excessive loss of NP cells caused by apoptosis disrupting the ECM homeostasis, which is closely related to ECM degradation (Zhang *et al*. 2021b). Therefore, inhibition of the inflammatory response and NP cell apoptosis may contribute to the prevention of ECM degradation and improve the progression of IVDD.

Traditional Chinese medicine has a long history in the prevention and treatment of IVDD (Zhu *et al*. 2020). For example, as a natural compound derived from olive leaves and oil, hydroxytyrosol played a protective role against IVDD and secondary neuropathic pain by inhibiting the secretion of TNF-α, IL-1β, and activation of NLRP3 inflammasome via inhibiting the NF-κB, PI3K/AKT, and ERK signaling pathways (Yu *et al*. 2022). Gong *et al.* showed that maltol, as a flavoring agent extracted from red ginseng, slowed IVDD development through the PI3K/AKT/NF-κB signaling pathway and NLRP3 inflammasome–mediated pyroptosis, which may be a viable medication for IVDD treatment (Gong *et al*. 2023). As an effective component of traditional Chinese medicine, ginsenoside Rg1 had been proved to promote ECM synthesis and cell proliferation, and inhibit apoptosis in degenerative NP cells via inhibiting the Wnt/β-catenin pathway, to alleviate the IVDD progress in the previous study (Yu *et al*. 2020). And Yang *et al*. (2022) also demonstrated that ginsenoside Rg1 had a significant inhibitory effect on the secretion level of inflammatory factors, redox activity, ECM degradation, and cell apoptosis in NP cells. However, the mechanism of ginsenoside Rg1 to alleviate the IVDD progression was far to be clear and comprehension. In the present study, ginsenoside Rg1 could improve the pathology of IVDD, inhibit the inflammation response and ECM degradation, and inhibit the activation of NF-κB signaling pathway in IVDD rats, to alleviate the IVDD progression.

A previous study demonstrated that IL-1β promoted inflammatory responses, and enhance the release of matrix-degrading enzymes in NP cells, indicating that IL-1β may play a critical role in the process of IVDD (Sun *et al*. 2018). So, IL-1β was used to induce NP cells to simulate IVDD environment in the present study, to explore the effect of ginsenoside Rg1 on NP cells during IVDD. Arctigenin, as the active ingredient of *Arctium lappa*, has been proved to suppress apoptosis, ECM degradation, inflammation, and activation of NF-κB pathway in NP cells induced by IL-1β via up-regulating miR-483-3p (Ji *et al*. 2022). Wang *et al*. (2022) suggested that kukoamine A was a bioactive component extracted from the root bark of *Lycium* and attenuated LPS-induced apoptosis, ECM, and inflammation in LPS-induced NP cells by activating the PI3K/Akt pathway. Emodin also was proved to protect NP cells against IL-1β-induced apoptosis and inflammation via inhibiting ROS-mediated activation of NF-κB pathway (Zhu *et al*. 2023). Ginsenoside Rg1 had been proved to promote growth and ECM synthesis of degenerative NP cells, delaying its degeneration (Lu *et al*. 2018). In the present study, ginsenoside Rg1 could promote cell proliferation and inhibit the apoptosis, inflammation response, and ECM degradation in IL-1β-induced rat NP cells, consistent with the results of Yang *et al*. (2022).

Accumulating evidence has highlighted that NF-κB is a key regulator of regulating cellular gene transcription, playing a significant role in biological processes such as inflammation and immune response, cell proliferation and apoptosis, and tissue damage and degeneration (Capece *et al*. 2022). In the IVDD process, inflammation oxidative stress and many other factors could induce matrix-degrading enzymes by activating NF-κB signaling pathway, accelerating the ECM catabolism of NP cells. Conversely, activation of NF-κB signaling pathway can increase the expression levels of many inflammatory mediators and chemokines, forming a vicious circle and further accelerating IVDD progression (Zhang *et al*. 2021a). So, NF-κB signaling pathway may be an ideal therapeutic target for IVDD. Sun *et al*. (2018) demonstrated that IL-1β and hypoxia synergetically contributed to the catabolic effects of NP cells by up-regulating the expression of matrix-degrading enzymes through the activation of NF-κB signaling pathway, indicating that the NF-κB signaling pathway is a key mediator of IVDD. Liu *et al*. (2021) also showed that Shikonin significantly inhibited the activation of NF-κB pathway to suppress the inflammatory response and apoptosis in LPS-induced NP cells. In order to investigate whether the NF-κB signaling pathway was involved in the regulation of ginsenoside Rg1 on the apoptosis, inflammation response, and ECM degradation of IL-1β-induced NP cells, the expression of NF-κB signaling pathway–related proteins was detected. As the results show, ginsenoside Rg1 inhibited the activation of NF-κB signaling pathway in the IL-1β-induced NP cells. So, it was speculated that ginsenoside Rg1 inhibited the apoptosis, inflammation, and ECM degradation in IL-1β-induced NP cells probably through inhibiting the activation of NF-κB signaling pathway.

## Conclusion

In summary, NF-κB signaling pathway might be related to the mechanism of action of ginsenoside Rg1 against IL-1β-induced apoptosis, inflammatory response, and ECM degradation in rat NP cells, and alleviate IVDD in rat. The conclusion suggested the ginsenoside Rg1 should be considered a potential new candidate for clinical therapy to attenuate IVDD.

### Supplementary Information

Below is the link to the electronic supplementary material.

Supplementary file1 (PDF 425 KB)

## Data Availability

The data were available from the corresponding author on reasonable request.
