# Supplementary material for: Ginsenoside Rg1 relieves rat intervertebral disc degeneration and inhibits IL-1β-induced nucleus pulposus cell apoptosis and inflammation via NF-κB signaling pathway
Source: In Vitro Cell Dev Biol Anim. 2024 Mar 14;60(3):287–99. doi: 10.1007/s11626-024-00883-6 (PMC11014818; doi:10.1007/s11626-024-00883-6)

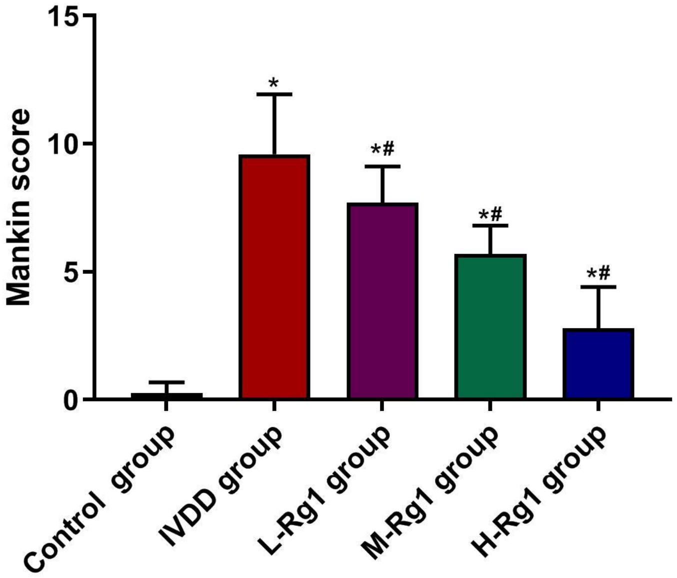

Figure 1 The modified Mankin score at IVD tissues in IVDD rats. Control group: n=8; IVDD group: n=10; L-Rg1 group: n=10; M-Rg1 group: n=10; H-Rg1 group: n=10. *, P<0.05, vs control group; #, P<0.05， vs IVDD group.

Animal : Western blot original picture

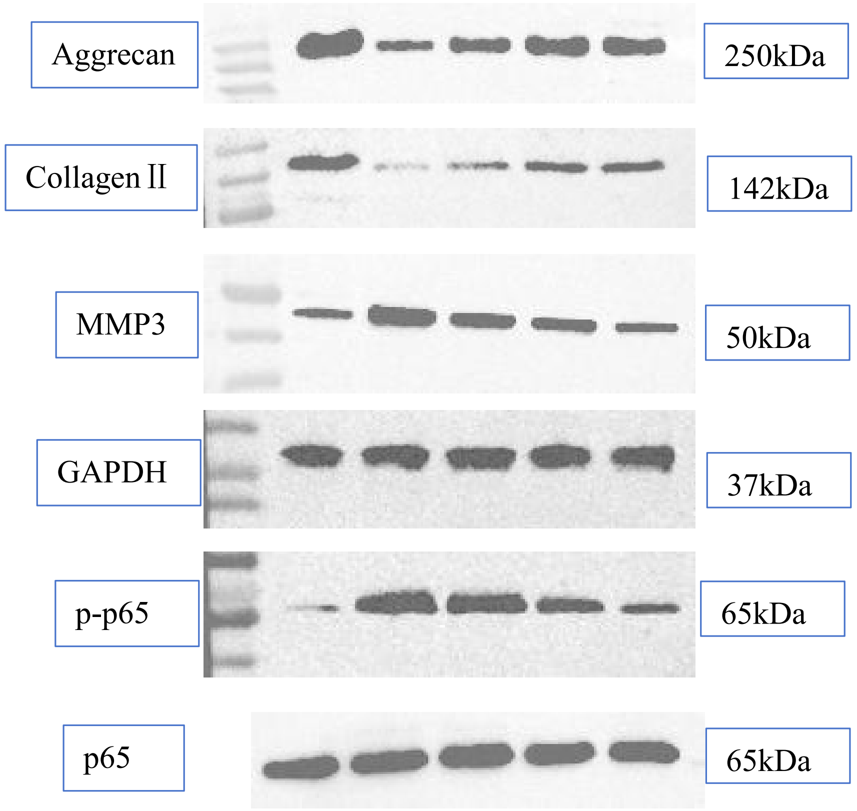

Cell : Western blot original picture

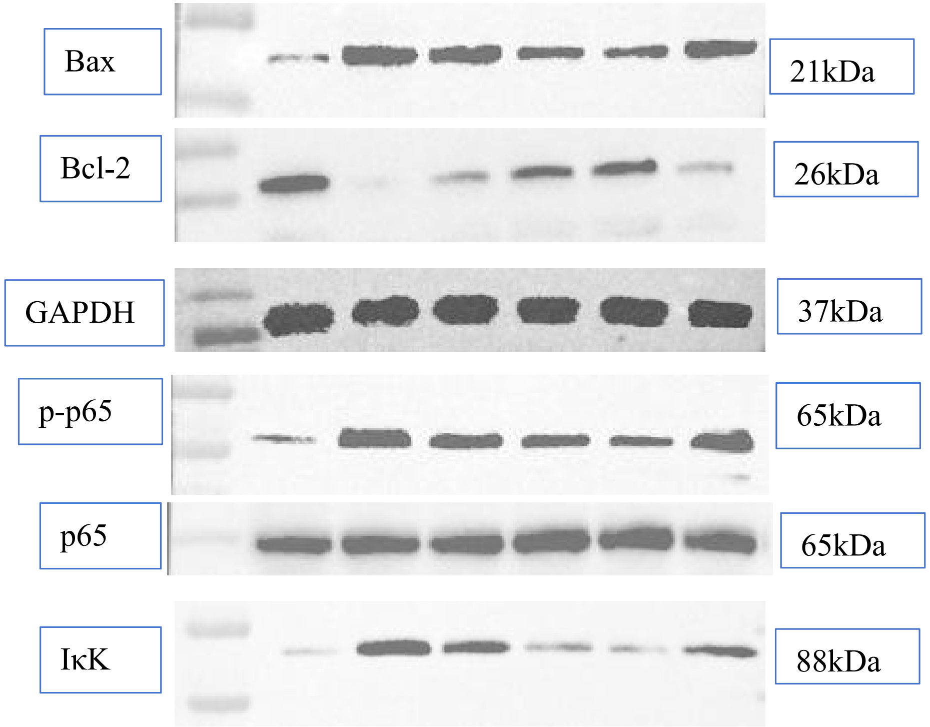

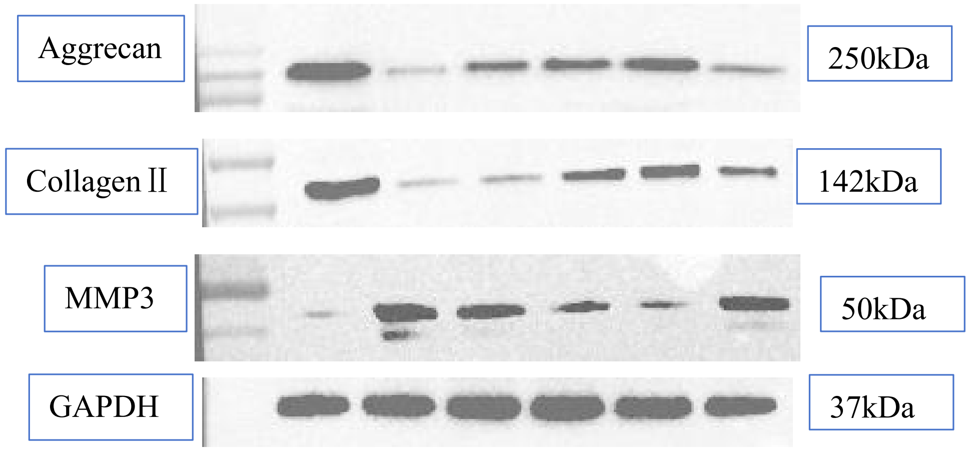
Aggrecan
250kDa
Collagen II
142kDa
MMP3
50kDa
GAPDH
37kDa

Figure 3B Control DAPI

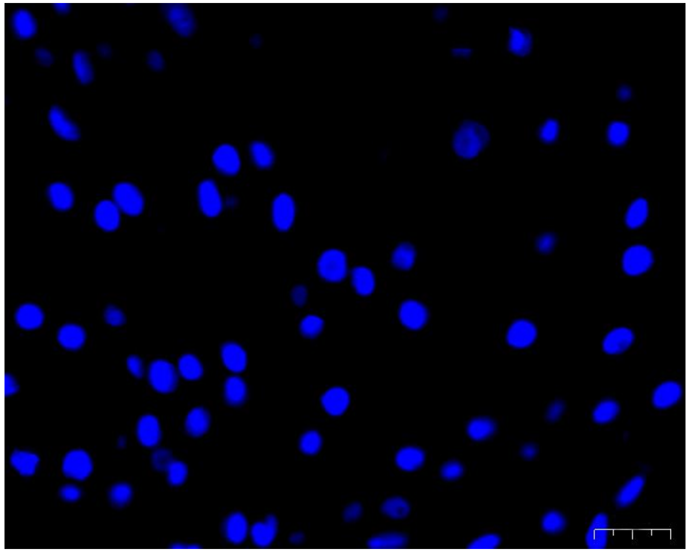

p65

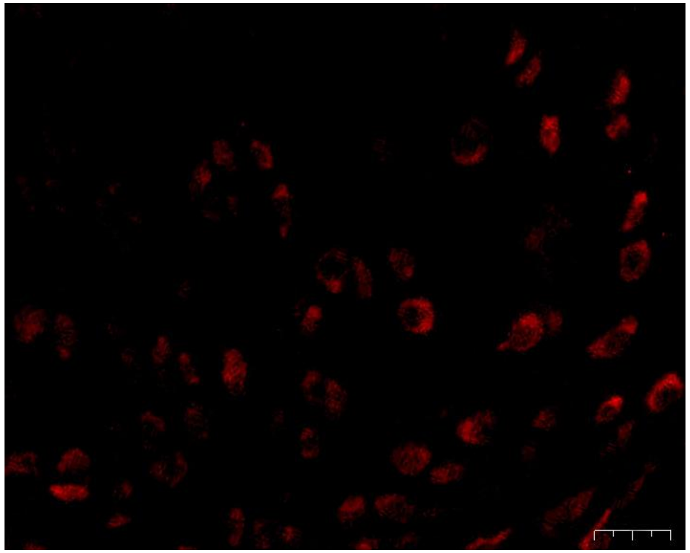

Merge

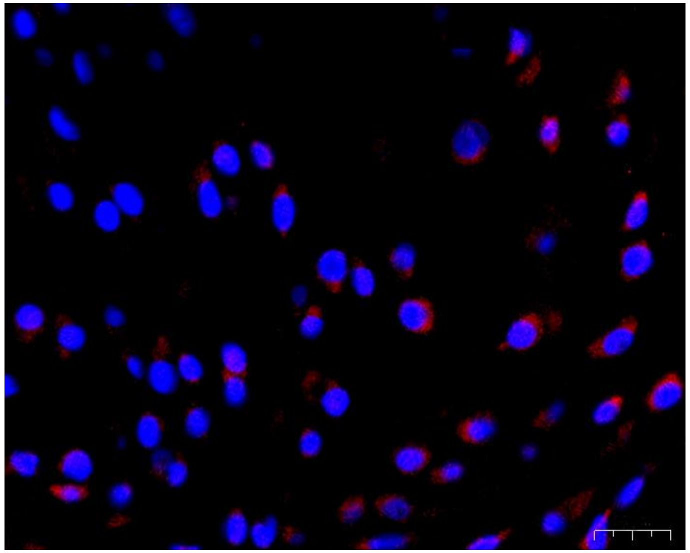

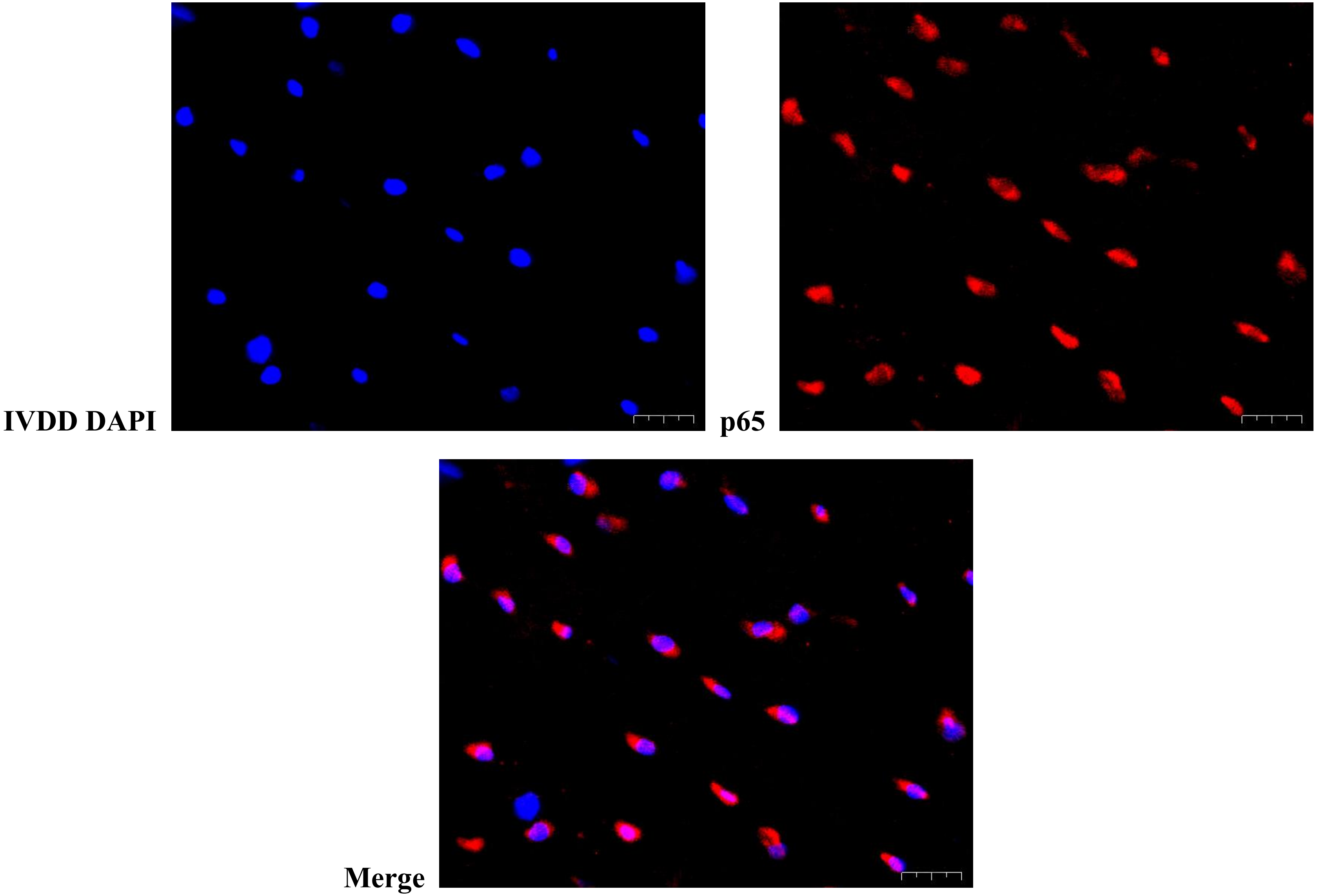
IVDD DAPI
p65
Merge

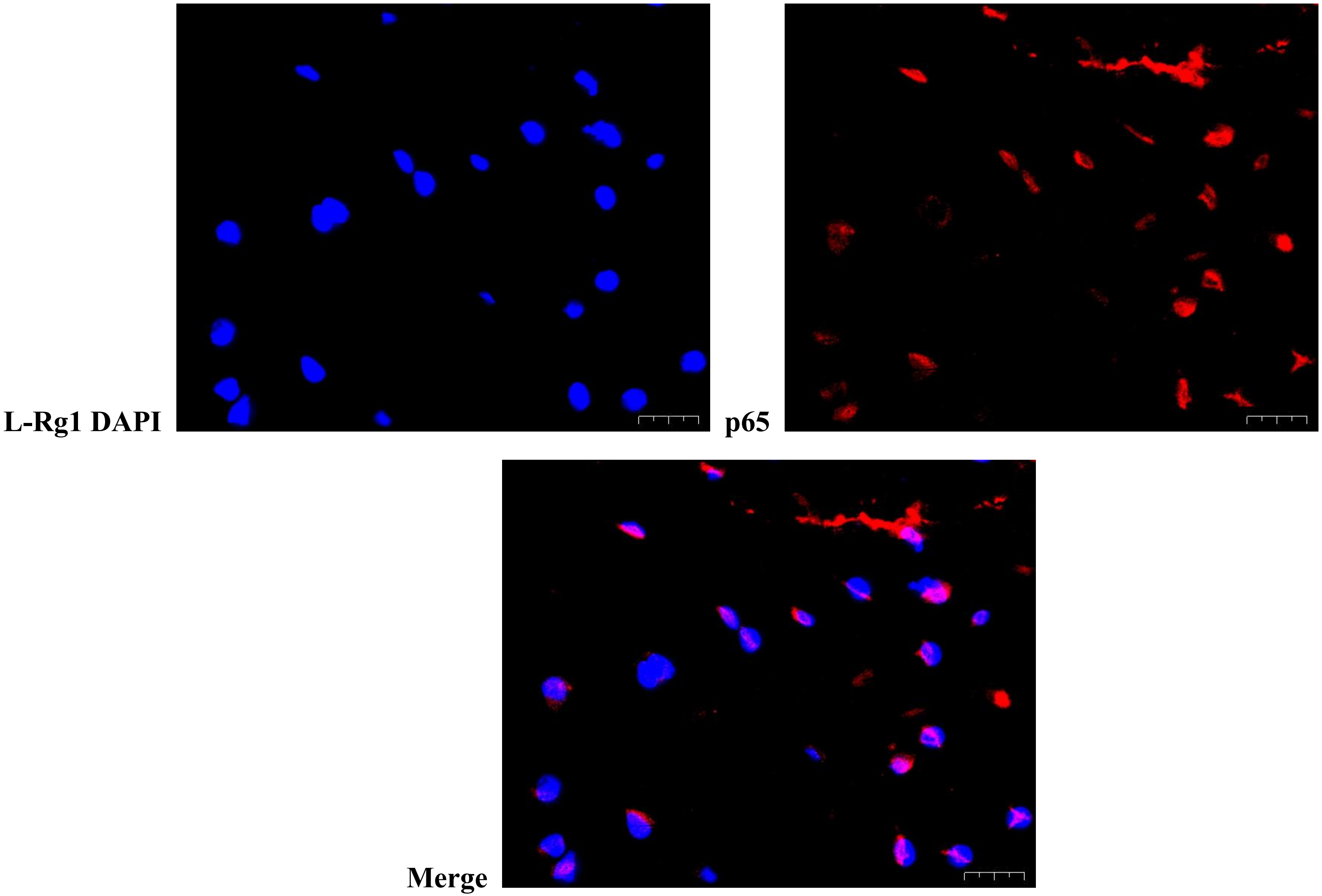
L-Rg1 DAPI
p65
Merge

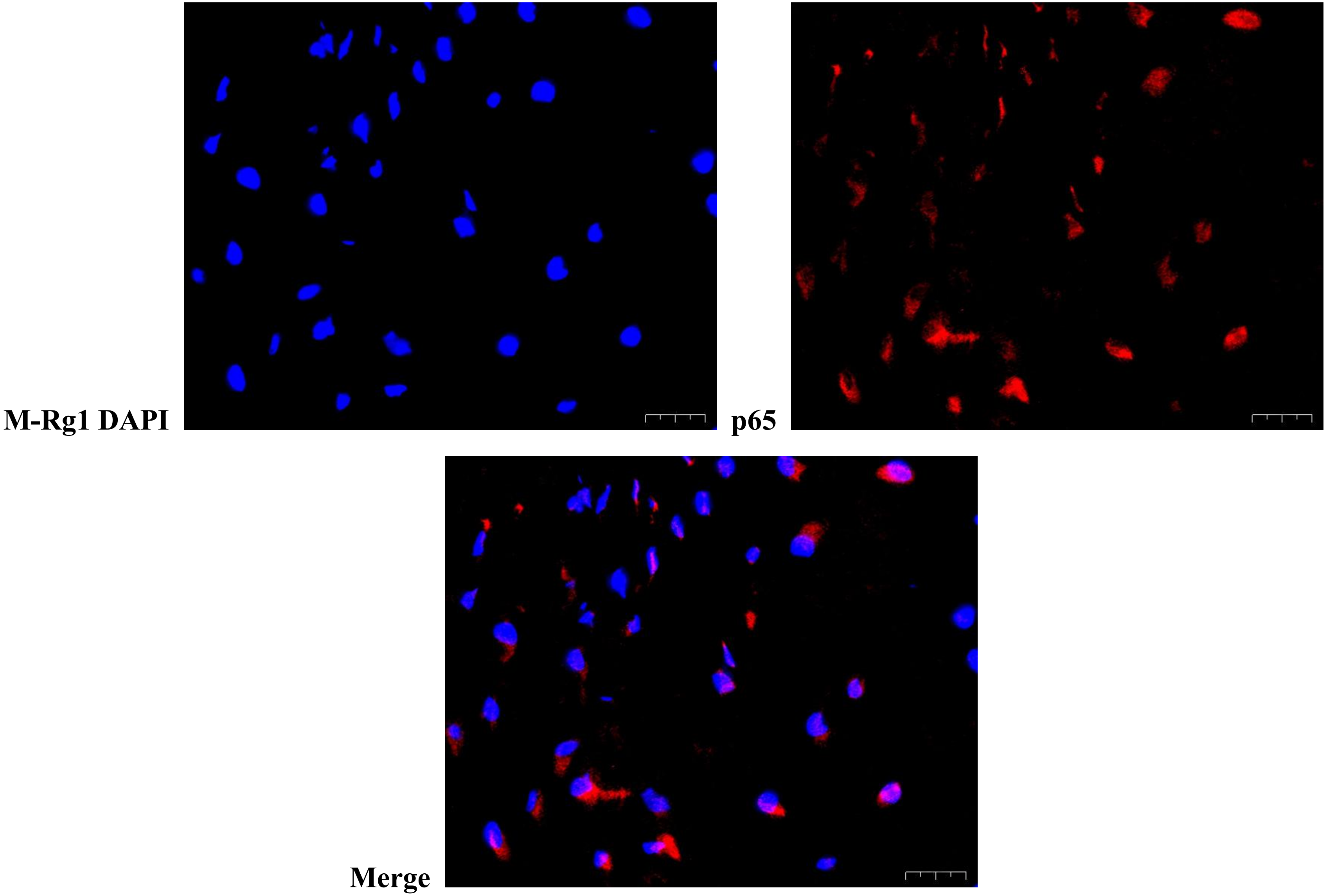
M-Rg1 DAPI
p65
Merge

H-Rg1 DAPI

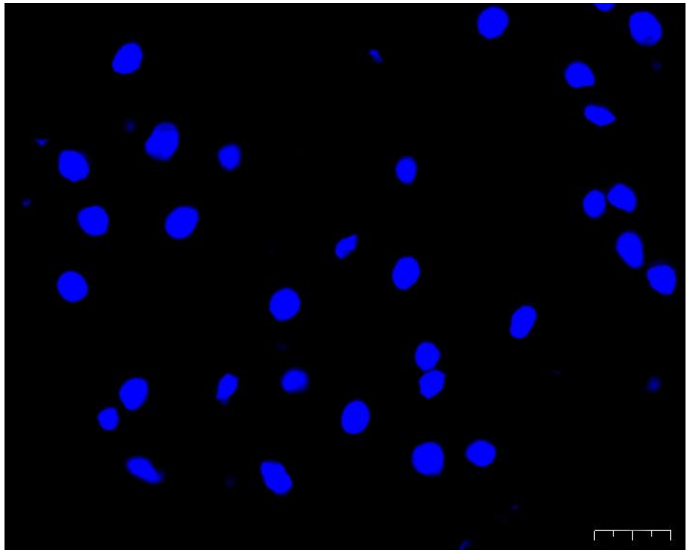

p65

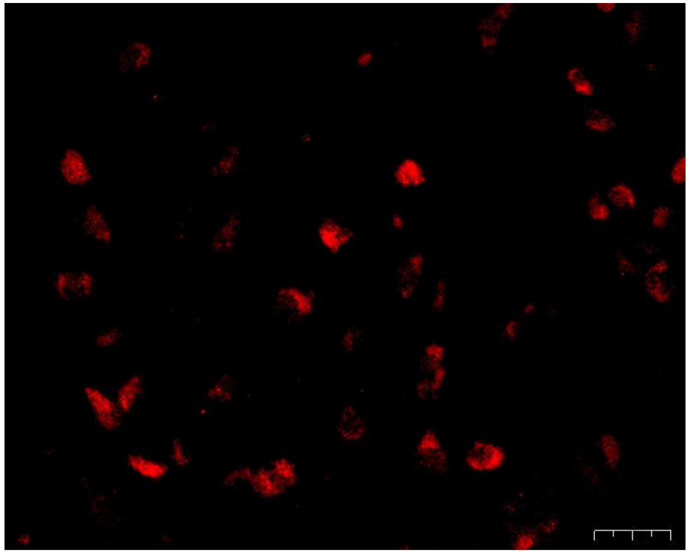

Merge

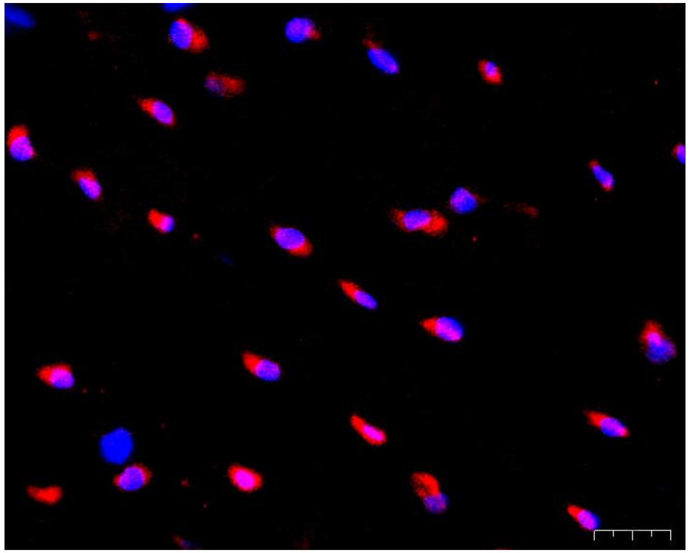

Supplement: Supplementary file 1 — Supplementary file1 (PDF 425 KB) [file 11626_2024_883_MOESM1_ESM.pdf]
